# INCORPORATING EXERCISE TO BUFFER AVERSIVE HEALTH EFFECTS OF LONELINESS AMONG OLDER ADULTS IN THE LIFE TRIAL

**DOI:** 10.1093/geroni/igae098.1176

**Published:** 2024-12-31

**Authors:** Navin Kaushal, Deepishka Pemmasani, Adrian Noriega de la Colina, Donya Nemati

**Affiliations:** Indiana University Indianapolis, Indianapolis, Indiana, United States; Indiana University Indianapolis, Indianapolis, Indiana, United States; McGill University, Montreal, Quebec, Canada; The Ohio State University, Columbus, Ohio, United States

## Abstract

**Introduction:**

In 2023 the US Surgeon General announced an epidemic of loneliness and isolation. Prolonged loneliness among older adults has been shown to predict dementia and cardiovascular disease (CVD). However, exercise has been demonstrated to have preventive effects on CVD and cognitive health, but its effects on buffering these outcomes manifested from loneliness, or gender effects have not been investigated. This study aimed to test two moderated-mediation models to investigate if exercise moderates the relationship between loneliness and health outcomes.

**Methods:**

The Lifestyle Interventions and Independence for Elders (LIFE) Study is a randomized controlled trial (n=1,600) that assigned older adults (aged 65+) to either an intervention or control group. The present observational study analyzed participants in the control group. Measures included: exercise (accelerometry), loneliness (Center for Epidemiologic Studies Depression Scale [CES-D]), CVD risk (handgrip test), and cognitive health (global cognitive function). Model #14 from Hayes PROCESS Macro 4.0 in SPSS was used to analyze the data.

**Results:**

In both models, females experienced greater loneliness compared to males(β=.25, p<.001). The CVD risk model found Moderate-to-Vigorous Physical Activity (MVPA) to independently predicted handgrip strength(β=.11, p<.001), and interacted with loneliness to predict handgrip strength(β=.05, p=.03). The cognition model also found MVPA to independently predict cognition(β=.14, p<.001), and interacts with loneliness to predict cognition(β=.07, p=.03), and also demonstrate total moderated-mediation effects(β=.02, 95%CI.003 to.367).

**Conclusion:**

Exercise can buffer aversive cardiovascular risk and cognition from loneliness. Lonely older adults are a high-risk demographic that should be sought for enrolling in exercise programs.

